# Cognitive Dysfunction following Cerebellar Stroke: Insights Gained from Neuropsychological and Neuroimaging Research

**DOI:** 10.1155/2022/3148739

**Published:** 2022-04-15

**Authors:** Qi Liu, Chang Liu, Yu Chen, Yumei Zhang

**Affiliations:** ^1^Department of Neurology, Beijing Tiantan Hospital, Capital Medical University, Beijing, China; ^2^China National Clinical Research Center for Neurological Diseases, Beijing Tiantan Hospital, Capital Medical University, Beijing, China; ^3^Department of Neurology, Memory and Aging Center, University of California, San Francisco, San Francisco, CA, USA; ^4^Department of Rehabilitation, Beijing Tiantan Hospital, Capital Medical University, Beijing, China

## Abstract

Although the cerebellum has been consistently noted in the process of cognition, the pathophysiology of this link is still under exploration. Cerebellar stroke, in which the lesions are focal and limited, provides an appropriate clinical model disease for studying the role of the cerebellum in the cognitive process. This review article targeting the cerebellar stroke population (1) describes a cognitive impairment profile, (2) identifies the cerebellar structural alterations linked to cognition, and (3) reveals possible mechanisms of cerebellar cognition using functional neuroimaging. The data indicates the disruption of the cerebro-cerebellar loop in cerebellar stroke and its contribution to cognitive dysfunctions. And the characteristic of cognitive deficits are mild, span a broad spectrum, dominated by executive impairment. The consideration of these findings could contribute to deeper and more sophisticated insights into the cognitive function of the cerebellum and might provide a novel approach to cognitive rehabilitation. The goal of this review is to spread awareness of cognitive impairments in cerebellar disorders.

## 1. Introduction

For many decades, the cerebellum has been considered a pure control machine for physical movement. However, several studies have shown the effect of the cerebellum on cognition and affect [1]. For the first time, Schmahmann et al. described a series of cognitive and behavioral dysfunctional symptoms in 20 patients with focal cerebellar lesions and summarized them with the term “cerebellar cognitive affective syndrome” (CCAS) [2]. The concept of CCAS is a milestone in the study of cerebellar cognition. In the past two decades, the role of the cerebellum in cognition has been widely verified in patients with different types of cerebellar disorders [3–5]. Cerebellar stroke, in which the lesion is confined to the cerebellum and not complicated by cerebral abnormities such as atrophy and hydrocephalus, provides an appropriate clinical model for studying the role of the cerebellum in the cognitive process. In addition, the distribution of cerebellar infarction lesions varied by different vascular territories, which was applicable to studying the cognitive topography of the cerebellum. Beyond physical disability, cognitive impairment has been recognized in cerebellar stroke patients, with approximately 64% of whom developed cognitive impairment and 24% of whom fulfilled diagnostic criteria for dementia [6].

In the current review, we aim to describe the cognitive profile following cerebellar damage, reveal the cognitive topography of the cerebellum, and explore the effect of the cerebral-cerebellar loop on cognition through the model of cerebellar stroke.

## 2. The Cognitive Deficit in Patients with Cerebellar Stroke

CCAS has been observed in patients with cerebellar infarction [7], which is characterized by executive function disturbances such as poor planning, perseveration of shifting set, abstract reasoning, and verbal fluency; visual-spatial disorganization and impaired visual-spatial memory; dysprosodia such as language difficulties, mild anomia, and agrammatism; and personality change characterized by a flattening or blunting affect, disinhibited, and inappropriate behavior [2].

### 2.1. Visual-Spatial Cognition

Spatial ability refers to the capacity to understand, reason about, and remember the spatial relations among objects or space. Botez described that a patient with a left superior cerebellar artery infarct did worse in picture arrangement, Benton's judgment of line orientation, and Hooper's visual organization test [8]. This is the first time visual-spatial deficits have been reported in isolated cerebellar infarction patients. Since then, several studies have noted impaired performance on a range of visual-spatial measures in patients with cerebellar stroke, including the block design tasks from the Wechsler Intelligence Scale [9, 10], copying and recall of Rey-Osterrieth complex figure [11], letter cancellation test [12], “blank clock” test [6], character-line bisection task [13], and mental rotation tasks [14]. These observations are suggestive of abnormalities in the field of visuoconstruction, visual attention, visuospatial planning, and the actualization of visual concepts. Furthermore, consistent with the contralateral connections between the cerebellum and the cerebral cortex, studies have found that patients with left cerebellar lesions are more likely to have deficits in visual-spatial tasks [15, 16] implying a possible link between lateralization of damage and spatial information processing.

### 2.2. Language

Early in the twentieth century, Holmes (1922) described the effects on the speech of cerebellar damage, a condition later to become known as ataxic dysarthria [17], which is commonly explained by the uncoordinated muscle movement of the articulatory organs. With the development of neurolinguistics tests, such as the Boston Diagnostic Aphasia Examination (BDAE) and the Aachener Aphasia test (AAT), high-level and subtle language impairment secondary to cerebellar damage began to be discovered gradually. Marien described a patient with ischemic infarction in the vascular territory of the right cerebellar superior artery (SCA). The patient presented with normal performance on standard neuropsychological tests and intact conversational skills but performed significantly poorer than norms in tasks of verbal fluency (phonemic and semantic), word stem completion, and oral naming speed [18]. By summarizing his symptoms, Marien proposed the concept of cerebellar-induced aphasia, which is characterized by nonfluent aphasia, including reduced speech initiation, decreased dynamics of language, word-finding disturbances, marked agrammatism, and reading and writing difficulties [18].

The evidence of language dysfunctions in cerebellar disorders comes from several case-series analyses and case reports. The disability in verbal fluency and semantic access are considered as prominent language symptoms following cerebellar infarction [19]. In word generation and retrieval tasks, patients had slowed language production and problems detecting their own error. Another common language symptom is grammatical impairment. Silveri (1996) reported a male following right cerebellar infarction presented motor aphasia for the first time [20]. The patient manifested sentence production deficit but did not reveal cortical abnormality that could account for the behavior [20]. The underlying mechanism remains to be elucidated, and experts supposed that the disruption of the connection between the cerebellum and frontal cortex contributes to language impairments in cerebellar infarction. Other language deficits including transcortical sensory aphasia [19], impaired reading and writing [6], spatial dysgraphia [21], and lexical-semantic retrieval functions of nonnative languages [22] were also revealed in stroke patients whose lesions were confined to the cerebellum.

### 2.3. Working Memory

Working memory (WM) is the ability that allows information to be maintained temporarily and manipulated online during diverse cognitive demands and is the central executive function. Previous studies have revealed the impaired function of WM in patients following an isolated cerebellar infarction [9, 23, 24]. Ravizza et al. investigated that selective damage in verbal WM occurred secondary to cerebellar disorders, but articulatory rehearsal strategies were unaffected, supporting motor problems did not implicate the impaired WM system [23]. WM comprises an attentional control system, a central executive, and two subsidiary systems for the storage of visuospatial and verbal material [25, 26]. By means of comparisons between two traditional and presumably less demanding “short-term memory” tasks, digit and word span, and the more recent and demanding listening span task, patients with cerebellar infarction only perform significantly worse in respect of the listening span task, suggesting impairment of the central executive domains of WM [9]. The result was consistent with a previous functional magnetic imaging study, which provided evidence that the cerebellum participates in an amodal bilateral neuronal network representing the central executive during working memory n-back tasks [27].

### 2.4. Executive Function

Executive functions, which define the ability to orchestrate different cognitive tasks to achieve a specific goal, are not a separate concept. These cognitive abilities are required for adapting to changes in the environment, for example, the capacity to plan, anticipate results, and focus resources appropriately to objectives, as well as the ability to keep attention for lengthy periods of time while distracted by adverse surroundings [28]. Five processes were distinguished in previous studies: attention and inhibition, task management, planning, monitoring the contents of working memory, and encoding [29, 30]. Executive deficits have been reported in a variety of studies of patients following cerebellar infarction, including effects on attention [6, 31], sequencing [32, 33], inhibition of inappropriate responses [34], task planning [6, 28], integration, and organization [35], using standard neuropsychological tasks such as reversed digit span, category switching, Trails A and B, Go/No-Go test, and the Stroop color-word interference.

The term “executive function” has long been used synonymously with the term “frontal lobe function.” [36] Executive disturbances are the most prominent symptom of CCAS. The ascending cerebellar projections to the frontoparietal cortex and the feedback loops may be the neural substrates of cerebellar involvement in the processing of execution.

### 2.5. Neuropsychiatric Features

In addition to language, executive, and visuospatial impairments, other psychological deficits following cerebellar disorders have been reported [37]. The list of cognitive functions that are impaired as a result of cerebellar dysfunction is expansive, including source memory [38], which is the ability to remember original contextual (i.e., temporal and spatial) features of an event or information, metalinguistics ability to understand metaphorical expressions or construct sentences with pragmatic quality [39], social cognition such as face emotion recognition [40], procedural learning [41], spatial-temporal confusion [42], and loss of emotions [43].

Cognitive impairments after isolated cerebellar stroke span a broad spectrum and are mild and transient [44]. A few studies conducted on the subacute or chronic period have not detected any significant deficit in cerebellar cognition [6]. Furthermore, cognitive disorders in cerebellar infarctions may recover in time, which means that the prognosis is good [45, 46].Traditional neuropsychological tests, which may detect well-defined cognitive profiles caused by supratentorial cerebral damage, are sometimes inadequately sensitive to identify “subclinical” abnormalities that might occur as a result of cerebellar diseases [47].The use of specific tests to detect CCAS may be critical in understanding cognitive changes following cerebellar disorders. The CCAS scale, which was developed in 2018, is an easily applicable bedside test to detect CCAS in clinical practice [48].The scale is a 10-item battery including significantly abnormal cognitive tests between patients and healthy controls: semantic fluency, phonemic fluency, category switching, verbal memory, digit span forward and backward, cube drawing, similarities, and Go/No-Go test. A pass/fail judgment is established for each test based on a threshold score, and one, two, or three and more failed subtests were defined as possible, probable, or definite CCAS, respectively. The latter study showed the area under the receiver operating characteristic (ROC) curves was 0.84 in isolated cerebellar infarction, indicating the strong diagnostic value of CCAS scale [49].

## 3. Cerebellar Lesion Location Determines Functional Deficits

Lesion-deficit studies in patients with focal cerebellar infarction provide pivotal insights into structure-function correlations. Studies found that lesion size was not associated with cognitive outcomes. Even very large lesions did not produce significant impairment in cognitive performance if they did not extend into the specific site of the cerebellum [14, 50]. This evidence hypothesis shows a strict localization of functions in the cerebellum. And a motor-cognitive dichotomy has been well-recognized: tasks with a significant motor component are impacted by a lesion in the anterior lobe, whereas performance on cognitive tasks with limited motor demands is more affected by lesions in the cerebellar posterior lobe regions [14, 51], suggesting an explanation of dissociation between motor deficits with preserved cognition and cognitive deficits without ataxia in patients with cerebellar disorders [42, 52]. Previous studies indicated that patients with posterior inferior cerebellar artery (PICA) lesions damaged cognitive function than those with superior cerebellar artery (SCA) lesions, which were manifested as motor dysfunction [10, 32]. There are other publications, however, which come to a different conclusion: no obvious differences in cognitive functions were found between patients with infarction of the PICA and SCA [12, 34]. Because the common SCA territory includes the anterior lobe as well as a portion of the posterior lobe, cognitive dysfunctions in SCA patients with extended posterior lesions may not be unexpected.

**Figure 1:**
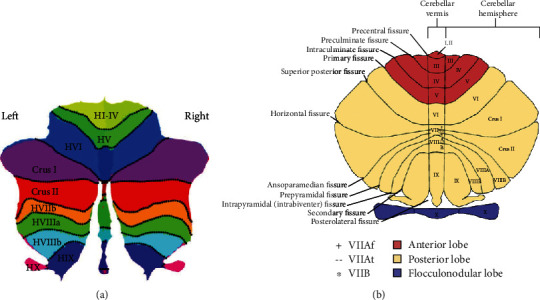
Flattened representation and illustration of the cerebellum and its major fissures, lobes, and lobules. Description: (a) Flattened representation of the human cerebellum developed by Diedrichsen et al. [105] In contrast to the vermis parts in the middle of the flat map, H stands for “hemispheric.” (b) The anterior lobe is colored red; the posterior lobe is cream and the flocculonodular lobe is purple. In the lobule VII, the VIIAf at the vermis expands in the hemisphere to become the Crus I. The lobule VIIAt at the vermis merges with the Crus II in the hemisphere, whereas the lobule VIIB retains its structural integrity both at the vermis and in the hemispheres. Author's diagram adapted from Schmahmann et al. [106] and first published in D'Mello et al. [107].

For the vague identification of the different cerebellar regions divided by vascular territory, medial/lateral, anterior/posterior, or vermis/hemisphere, Larsell et al. (1972) constructed a detailed atlas of the human cerebellum [53]. The atlas divided the human cerebellum into ten regions marked with Roman numerals from the anterior (regions I-V) to the posterior (regions VI-X), which demonstrates the details of the cerebellar cortex within the three cardinal planes in Talairach proportional stereotaxic space and provides a more contemporary and accessible cerebellar nomenclature (Figure 1). Damage to regions VI and VII has been observed to be associated with impaired cognitive performance [51], matching the functional topography of the cerebellum in healthy controls using task-based and resting-state functional magnetic resonance imaging (fMRI) [54–57].

### 3.1. The Theory of Universal Cerebellar Transform

**Figure 2:**
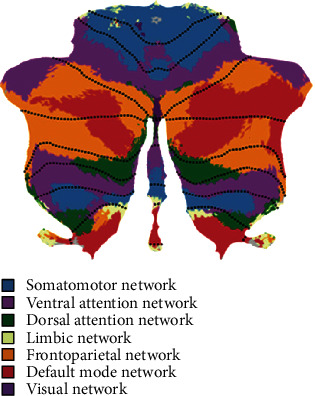
A map of the human cerebellum based on functional connectivity to seven major networks in the cerebrum. Description: Author's diagram adapted from Schmahmann et al. [108] and first published in Buckner et al. [61]

The theory of universal cerebellar transform (UCT) indicates that the cerebellum has a consistent internal structure and works as a modulator to optimize performance according to context [58]. The strict localization of functions in the cerebellum results from the heterogeneity of cerebellar connections with extracerebellar structures rather than variations in the cerebellar microstructure itself [59]. Functional neuroimaging studies confirmed that the cerebellum has extensive connectivity with different cerebral areas: the cerebellar posterior lobe connects with the prefrontal, posterior parietal, superior temporal, and limbic cortices, while the anterior lobe connects with the primary motor and premotor cortex [15, 60–62].Furthermore, resting-state functional connectivity analysis shows that the cerebellum can be divided into elaborate functional regions based on the patterns of anatomical connectivity between different regions of the cerebellum and association areas of the cerebral cortex [15, 56, 63] (Figure 2). Various psychological deficits following different cerebellar lesions are assumed to be a result of the interruption of different cerebro-cerebellar cognitive loops: prefrontal cortical associated cerebellar areas in relation to executive control, parietal cortical areas with respect to visuospatial function, and frontotemporal regions in relation to linguistic function.

### 3.2. Functional Topography of Cerebellum

Voxel-based lesion-symptom mapping (VLSM) is an imaging method that analyzes the relationship between brain lesions and behavioral performance on a voxel-by-voxel basis [64]. This method offers better resolution than grouping stroke lesions by the affected artery or conducting region of interest analyses. With the use of VLSM, cerebellar functional topography can be described well in the disease model of cerebellar stroke for its confined lesions.

**Figure 3:**
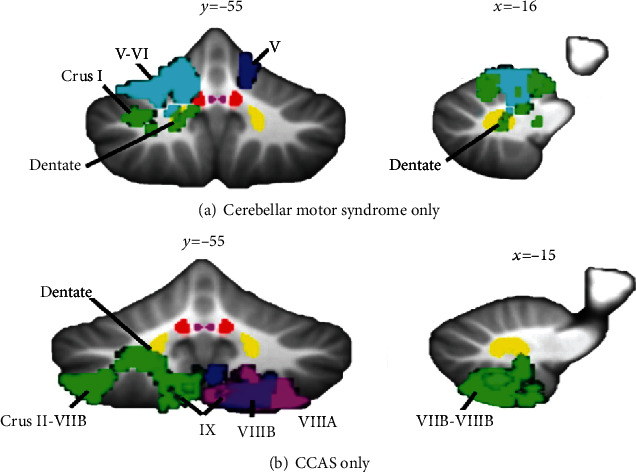
Lesion symptom mapping in patients with cerebellar infarction. Description: (a) Lesions of lobules IV–V of the anterior lobe extending into adjacent lobule VI produce the cerebellar motor syndrome of ataxia but not cerebellar cognitive affective syndrome (CCAS). (b) Lesions confined to posterior lobe lobules Crus II through lobule IX produce the cerebellar cognitive affective syndrome but no motor ataxia. Different colors represent the lesions of individual patients. Author' s diagram developed by Stoodley et al. [51].

Consistent with traditional group analyses, the study of VLSM further confirmed damage in the posterior lobe, especially in the region of VI and VII that produced the cognitive impairment [12, 49, 51]. Richter et al. firstly used this technique in patients following isolated cerebellum infarction to reveal functional regions underpinning cognition in the cerebellum and demonstrated that impaired performance in a verbal fluency task was associated with the lesion of the right hemispheric region Crus II [12].Stoodly et al. (2016) found patients with damage to cerebellar lobules III–VI had worse ataxia symptoms, while posterior cerebellar damage involving lobules VII and VIII was a risk factor for cognitive deficits, which further validated the anterior-sensorimotor/posterior-cognitive dichotomy in the cerebellum (Figure 3). In addition, different locations of lesions were found to lead to significantly poorer scores on particular cognitive tasks, such as language (right Crus I and II extending through IX), spatial (bilateral Crus I, Crus II, and right lobule VIII), and executive function (lobules VII–VIII) [51].Chirino-Pérez et al. recently conducted a support vector regression-based multivariate VLSM study in 22 patients with chronic isolated cerebellar strokes and used the CCAS scale to detect cognition damage more sensitively. They found global cognition impairment was associated with damage to the right lateral posterior lobe of the cerebellum, particularly in region VI and Crus I [49]. The subanalyses of this study also revealed that semantic fluency, category switching, and cube drawing were impaired severely when damage was involved right VI, VIIb, Crus I, and Crus II [49].

In addition, some other studies of VLSM using specific cognitive tasks focused on exploring a link between specific cognitive impairment and lesion location in patients with cerebellar infarction. Those studies observed that impaired phonemic fluency correlated with lesions in right Crus II, IX, and X and the deep nuclei; [65] visual attention deficit correlated with lesions of the pyramid of the vermis, the culmen, and partly the inferior semilunar lobule; [66] visuomotor rotation adaptation damage correlated with lesions in region VI; [67] impaired spatial and temporal visual attention correlated with the left posterior cerebellar region Crus II; [31]and difficulties in the recognition of emotion from voices (emotional prosody) correlated with lesions in the right region VIIb and VIII, and Crus I and II [68, 69]. More details are provided in Table 1. Studies of VLSM make an effort to identify cerebellar regions which are crucial to the presence of cognitive dysfunction and describe a precise cerebellum cognitive functional topography. The conclusion of those studies is consistent with studies on healthy subjects [54, 70].

## 4. Cerebro-Cerebellar Loop: Evidence from Neuroimaging

The cognitive deficits following cerebellar damage were held to result from the disrupted connectivity between the posterior cerebellum lobe and cerebral association areas [71]. Anatomic and imaging studies have indicated that the cerebro-cerebellar loop consists of afferent inputs through cortico-ponto-cerebellar projections and an efferent pathway through the cerebello-thalamic-cortical [72]. At the cellular level, there exists a direct excitatory loop from the cortex through the cerebellar nucleus dentatus and an inhibitory input through the Purkinje cells in the cerebellar cortex.

Studies employing fMRI in cerebellar stroke patients further provide evidence for a cortico-cerebellar connection as the functional substrate of cognition. Ziemus et al. explored the activation pattern changes during an n-back working memory task in patients with isolated cerebellar infarct: compared with healthy controls, bilateral increased BOLD activations in the ventrolateral prefrontal cortex, dorsolateral prefrontal cortex, parietal cortex, presupplementary motor area, and anterior cingulate were found in cerebellar patients during the task [35]. Wang et al. revealed that the abnormal alterations in the right posterior cingulate gyrus, bilateral median cingulate and paracingulate gyri, and right precuneus may play a core role in the cognitive impairment following cerebellar infarctions using diffusion tensor imaging [73]. Fan et al. found that the lower fractional amplitude of low-frequency fluctuation in the left hippocampus and right cingulate gyrus is related to poor cognitive performance in patients with acute posterior cerebellar infarction [74].

### 4.1. Crossed Cerebello-Cerebral Diaschisis and Lateralization

Crossed cerebello-cerebral diaschisis (CCD) has been described in patients with focal cerebellar lesions showing decreased cerebral perfusion and metabolism contralateral to cerebellar lesions [75].The cellular and molecular events of Wallerian degeneration that spread over the cerebello-cerebral tracts distant from the primary cerebellar lesions are speculated to be a possible mechanism for the phenomenon [76, 77]. Functional neuroimaging studies using single photon emission computed tomography (SPECT), positron emission tomography (PET), and near-infrared spectroscopy have demonstrated that cerebral hypometabolism and hypoperfusion may contribute to cognitive dysfunction in cerebellar infarction [8, 18, 78–81]. A quantified SPECT study showed that, in the absence of any structural damage in the supratentorial brain regions, contralateral hypoperfusion in the left medial frontal lobe secondary to the cerebellar lesion may explain frontal lobe symptoms such as executive dysfunction, apathy, and disinhibition [79].

For the crossing of cerebello-cortico-cerebellar connections, the functional lateralization of cerebellar disorders is currently being considered. Clinical studies showed patients with cerebellar infarction lesions in the right posterior lobe manifested poorer cognitive performance than in left-lateralized regions [49, 50].In addition, studies also indicated the cognitive characteristics of different cerebellar hemispheric infarcts: language dysfunctions often follow damage to the right cerebellar hemisphere, whereas visual-spatial disability can result from left cerebellar hemisphere lesions [51, 82]. However, the side of the lesion showed no significant effect on cognitive performance in other studies [12, 32]. Two possible reasons may help explain this inconsistent conclusion. One hypothesis is that, as bilateral cortical activation was observed during linguistic and spatial tasks [83, 84], cerebral cortex functions are not always completely lateralized. An alternative interpretation is that cerebellar reserve, the capacity of the cerebellum to compensate for tissue damage or loss of function by the formation of new synaptic connections with cerebral cortical neurons [85], can prompt changes in connectivity between distinct networks and lead to reorganization of cerebellar functional topography [86].

## 5. Theory of Cerebellar Cognitive Function

Dysmetria of thought (DoT) is proposed as a fundamental framework attempting to explain the cognitive symptoms in patients with focal cerebellar lesions [87]. The intact cerebellar function facilitates actions harmonious with the goal, appropriate to the context, and judged accurately and reliably according to the strategies mapped out prior to and during the behavior. It is hypothesized that the prefrontal discharges are regulated and modulated rather than generated, by cerebellar structures. Cognitive dysfunctions following cerebellar disorders are associated not with the death of cortical neurons but with “discordance” in their operation, which explains why cognitive impairments after focal cerebellar disorders in adults are mild or transient.

Other theories which are compatible with the DoT theory on how the cerebellum modulates cognitive function have been inspired by anatomical and physiological studies. These include the theories of biological clock [88], timing machine [89], error detection [90], sequence learning [91], automatization [92], dynamic state monitoring [93], neuronal machine [94], and implementing supervised learning using computational and engineering organizational principles [95].

## 6. Future Perspective

CCAS identifies the key characteristics of cerebellar patients' cognitive and emotional impairments. Aside from deficits of executive functions, visuospatial cognition, linguistic functions, and personality changes in cerebellar disorders, the involvement of the cerebellum in metalanguage and social cognition has also been discovered [39, 40]. Metalinguistic abilities include explicit awareness of abstract language representations. Patients with cerebellar lesions may have defects in perceiving ambiguities, formulating intelligible statements for a specific context, inferring logically, and comprehending figurative language, in contrast to the grammatical and semantic abilities that have been retained [39]. Social cognition is the process of observing and understanding the behavior and mental state of others, including oneself, in response to nonverbal or verbal stimuli [96]. Distortions in social cognition are usually regarded as the underlying dysfunction causing severe malfunctions in social and affective function. Mirroring, mentalizing, and abstract judgment are three sets of categories of social cognition. According to an activation likelihood estimation meta-analysis, the cerebellum is not responsible for any specific function but rather increases the efficiency with which other neocortical regions accomplish their own processes [97]. The role of the cerebellum in metalanguage and social cognition is consistent with the unifying framework of the UCT and the DoT and provides new insights into the nature of the cognitive impairments in patients with the CCAS.

Besides the cerebellar functional topography mentioned above, a recent resting-state imaging analysis revealed novel functional properties of cerebellar double motor representation (lobules I-VI and VIII) and triple nonmotor representation (lobules VI/Crus I, Crus II/VIIB, and IX/X) [63]. The first cerebellar motor representation that targets the primary motor cortex is engaged in motor control, whereas the second cerebellar motor representation that targets regions surrounding the precentral gyrus is more important for movement planning rather than movement execution [55]. The three cognitive representations are considered in the same way: the relationship between the first and second motor representations is similar to that of the first/second and third cognitive representations [57, 63]. And future studies are needed to explore what different functions are enabled by the different cognitive representations and what the differing consequences of lesions to the different representations are. Also, experts indicated that functional subdivisions did not align with lobular borders, which are often utilized to summarize functional data; they suggested that the novel parcellation serves as a functional atlas should be performed for future neuroimaging research [56]. Due to the localization and diversity of the lesions, cerebellar stroke could be a good disease model to demonstrate a more detailed and specific cerebellar functional topography by using fMRI techniques.

Due to the role in motor and nonmotor function, the cerebellum is attracting scientists interested in basic and clinical research of neuromodulation. Transcranial direct current stimulation (tDCS) and repetitive transcranial magnetic stimulation (rTMS) of the cerebellum can modify cognitive function, and targeted stimulation to narrow areas within the cerebellum produces differential effects on cognitive tasks such as language, memory and learning, and visuospatial orientation [98, 99]. Previous research has confirmed the validity of cerebellar noninvasive stimulation. A previous study reported that a patient with a left cerebellar stroke showed improvement on tasks modeling procedural learning after administration of rTMS to the unaffected right cerebellar hemispheres [41]. In addition, cerebellum-targeted rehabilitation exercises have provided a realistic opportunity for intervention in mental diseases such as autism spectrum disorders, affective disorders, and psychotic spectrum disorders, as well as Alzheimer's disease and aphasia [100, 101]. However, because of the highly convoluted nature of the cerebellar cortex, effects of noninvasive cerebellar brain stimulation are difficult to anticipate, and the robustness and replicability of previous findings will need to be evaluated before any recommendations on these forms of therapy can be made [102]. More research should be done to standardize the stimulation paradigms of cerebellum-targeted brain stimulation [103, 104].

## 7. Conclusion

Cerebellar involvement in cognition has long been a research topic but is gaining increasing clinical attention. This review provides new details about how cognitive dysfunctions manifest in cerebellar stroke. Evidence from neuroimaging and patient populations suggests that the posterolateral cerebellum contributes to cognitive processing and demonstrates a detailed functional topography using VLSM. In addition, the disruption of the cerebro-cerebellar loop has been considered as the mechanism of CCAS. The application of the tools of contemporary cognitive neuroscience may allow us to understand the cerebellum's role in cognition and emotion more in-depth. And new opportunities may also be possible for rehabilitation intervention in neuropsychiatry by targeting focal areas in the cerebellar node of the distributed cerebro-cerebellar networks subserving human cognition and emotion.

## Figures and Tables

**Table 1:** Voxel-based lesion-symptom mapping studies of cognitive function in patients following cerebellar infarction.

Number	Studies	Scanner	Subjects	Task/clinical performance	Result
1	Richter (2007)	3T	21 patients vs. 25 controls	Verb generation task, neglect tests (letter cancellation, line bisection), visual extinction test, verbal fluency task	Impaired performance in the verbal fluency task correlated with lesions in the right region Crus II
2	Baier (2010)	3T	26 patients vs. 15 controls	Covert visual attention task	Impaired covert visual attentional processes correlated with lesions in vermal structures such as the pyramid
3	Stoodley (2016)	3T	18 patients vs. norms	Wechsler Adult Intelligence Test-3, Trails A and B, Wisconsin Card Sorting Task, Wechsler Memory Scale, fluency task, Boston Naming Test, Benton Judgment of Line Orientation, mental rotation and Rey figure task	Cognitive impairment correlated with lesions in posterior lobe. More specifically, lesions of right Crus I and II extending through IX lead to poorer scores on language, lesions of bilateral Crus I, Crus II and right region VIII associate with spatial, and lesions of region VII–VIII associate with executive function
4	Kim (2017)	3T	24 patients vs. norms	Geriatric Depression Scale	Lesions in left VI, VIIb, VIII, Crus I, and Crus II are related with severity of depressive symptoms
5	Thomasson (2019)	3T	15 patients vs. 15 controls	Emotional prosody recognition task	Emotional misattributions correlated with lesions in right region VIIb, VIII and IX; and rhythm discrimination correlated with lesions in region VIIb
6	Pérez (2021)	3T	22 patients vs. 22 controls	Montreal Cognitive Assessment, cerebellar cognitive affective syndrome scale(CCAS-s)	Lesions in right region VI and Crus I are related with poor performance of CCAS-s, semantic fluency subtest, and cube drawing subtest; lesion in right region VIIb, Crus I, and Crus II are related with poor category switching score
7	Craig (2021)	1.5 T	14 patients vs. 24 controls	Reflexive and voluntary covert attention task, attentional blink task, sustained attention to response task	Deficits in spatial and temporal visual attention correlated with lesions of left Crus II
8	Thomasson (2021)	1.5T	24 patients vs. 24 controls	Emotional prosody recognition task	Emotional misattributions correlated with lesions in the right region VIIb, VIII, Crus I and II
